# Evaluation of Semantic Segmentation Performance for a Multimodal Roadside Vehicle Detection System on the Edge

**DOI:** 10.3390/s25020370

**Published:** 2025-01-10

**Authors:** Lauren Ervin, Max Eastepp, Mason McVicker, Kenneth Ricks

**Affiliations:** Electrical and Computer Engineering Department, The University of Alabama, Tuscaloosa, AL 35487, USA; mteastepp@crimson.ua.edu (M.E.); mmcvicker@crimson.ua.edu (M.M.); kricks@eng.ua.edu (K.R.)

**Keywords:** semantic segmentation, CNN, LiDAR, camera, roadside detection system, inference, deep learning, trucks, trains, embedded platforms

## Abstract

Discretely monitoring traffic systems and tracking payloads on vehicle targets can be challenging when traversal occurs off main roads where overhead traffic cameras are not present. This work proposes a portable roadside vehicle detection system as part of a solution for tracking traffic along any path. Training semantic segmentation networks to automatically detect specific types of vehicles while ignoring others will allow the user to track payloads present only on certain vehicles of interest, such as train cars or semi-trucks. Different vision sensors offer varying advantages for detecting targets in changing environments and weather conditions. To analyze the benefits of both, corresponding LiDAR and camera data were collected at multiple roadside sites and then trained on separate semantic segmentation networks for object detection. A custom CNN architecture was built to handle highly asymmetric LiDAR data, and a network inspired by DeepLabV3+ was used for camera data. The performance of both networks was evaluated, and showed comparable accuracy. Inferences run on embedded platforms showed real-time execution matching the performance on the training hardware for edge deployments anywhere. Both camera and LiDAR semantic segmentation networks were successful in identifying vehicles of interest from the proposed viewpoint. These highly accurate vehicle detection networks can pair with a tracking mechanism to establish a non-intrusive roadside detection system.

## 1. Introduction

As transportation infrastructure continues to grow, there is an increasing need to detect target vehicles in an unobtrusive manner for logistics applications. In densely populated cities, many roadways are already equipped with traffic cameras that could be used for vehicle detection. However, there are a number of back roads, gravel roads, and railways where cameras are not always present that many large vehicles traverse when transporting equipment. These routes are frequently traveled to provide deliveries to more remote locations. In these situations, new sensor installments are required for non-contact vehicle detection. Although physical trackers for vehicles exist, they can become costly when deployed at a large scale, and they first require access to the targets of interest [1]. Thus, an alternative, non-intrusive approach is to identify the vehicles from a roadside view with the use of vision sensors such as a LiDAR or camera. A single roadside vehicle detection system can be set along a route to track an infinite number of targets, no matter how remote the path may be. Since this system is portable, it can also change locations as traveled routes are updated or new targets are identified. When changing views, additional training may be required to maintain high detection accuracy. To track the number of targets crossing a route, the detection networks can be integrated into a larger tracking system that identifies individual target instances over time.

The first step of developing this system is identifying data from a roadside view for training and testing. There are very few publicly available and robust roadway datasets which consist of multimodal data in a range of environmental conditions, let alone from a roadside perspective [2]. Datasets that target roadways or vehicles usually do so from two different viewpoints: first person or overhead. These datasets are primarily used for autonomous driving or traffic monitoring systems [3]. The first person view is captured from sensors either within or mounted atop a vehicle [4,5,6,7,8,9]. These datasets train models with constantly changing viewpoints and targets with varying distances from the sensors. Additionally, many of these datasets with changing viewpoints comprise complicated scenes with twenty or more different classes. The proposed roadside detection system is only interested in a single target of interest—train cars when targeted on railways and semi-trucks when targeted on roads—and is thus a simpler problem to solve. This can lead to lower rates of inference mislabeling in practice. Another view commonly used in the literature is an overhead view that is captured either from traffic cameras or sensors on a UAV above the roadway [10,11]. Similar to a roadside system, the statically mounted traffic cameras maintain a set distance from targets of interest. These Bird’s-Eye-View systems may not be well suited where transportation infrastructure is not already setup or non-urban areas with heavy occlusion zones. Conversely, a deployable roadside vehicle detection system is easy to relocate to avoid natural or changing occlusions such as foliage or large, manmade structures.

There are also publicly available datasets involving train cars for further logistics tracking, but they have their limitations. They are normally in a first person view from the train car targeting the tracks [12,13,14]. The main purpose of these datasets is to improve train safety, rather than to track cargo [15]. We offer a unique roadside perspective for evaluating object detection of both train cars and semi-trucks using computer vision. Semantic segmentation performance is evaluated and compared for camera and LiDAR data. This approach yields successful semantic segmentation results suited for situations where other vantage points are not possible or preferable for sensor deployment.

**Figure 1:**
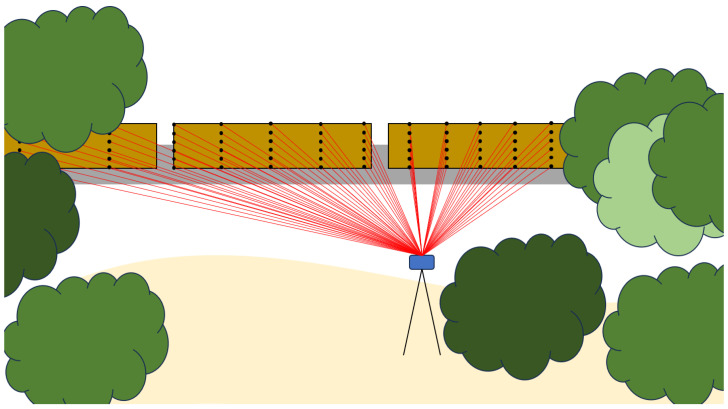
The proposed portable roadside vehicle detection system is depicted. All LiDAR and camera sensors, represented by the blue rectangle, are mounted to the side of a road/railway/path. From this viewpoint, the sensor scanlines are able to hit passing targets of interest, e.g., train cars highlighted in tan, on a known path.

We utilize the UA_L-DoTT dataset, which features a large set of multimodal, labeled data collected from this unique roadside perspective [16]. This dataset comprises 93,000 images from a monocular camera and 350,000 point clouds from multiple LiDARs with varying operational characteristics collected under changing weather and lighting conditions. An example of the sensor setup targeting one of the railway sites is displayed in Figure 1. To our knowledge, no other dataset targeting train cars and semi-trucks with these parameters exists. Due to the heterogeneous data, two separate Convolutional Neural Network (CNN) architectures were developed and trained to evaluate semantic segmentation performance for classifying vehicles of interest. All code is publicly available on the UA Roadside Semantic Segmentation GitHub Organization (https://github.com/UA-Roadside-Semantic-Segmentation/Multimodal-Roadside-Detection/tree/main, accessed on 1 May 2024). The mean Intersection-Over-Union (mIOU) and standard deviation metrics were calculated for both camera- and LiDAR-based models on training hardware. mIOU is commonly used to evaluate the accuracy of a model’s prediction. The standard deviation is included as a metric to give insight into the number of false positives and negatives present in each system. Semantic segmentation inference was also performed on embedded platforms suitable for edge deployment at a roadside view for real-time train car and semi-truck monitoring.

Our results support many of the inherent advantages provided by LiDAR, and they indicate that the segmentation performance of LiDAR systems is comparable to that of camera systems. Specifically, the LiDAR models have comparable validation and testing mIOU to the camera model. The LiDAR models typically had a higher frame rate and power consumption than the camera model on the embedded platforms. The LiDAR models also significantly outperformed the camera model in low-light conditions, and the generic LiDAR model performed well across data from multiple different LiDARs, indicating that sensor-agnostic deployments can perform favorably. However, the results indicate that the LiDAR models do not generalize as well as the camera model, and thus could require additional training for comparable performance when deployed to new locations.

The remainder of the paper is organized as follows. The Related Works section discusses the relevant and recent contributions to the semantic segmentation problem with both camera and LiDAR data. Within the Materials and Methods section, the location of the large dataset of collected camera images, LiDAR point clouds, and correlating data are provided in the Dataset subsection. The details of the CNN architectures used for semantic segmentation are covered in the Camera Architecture and LiDAR Architecture subsections. The breakdown of the training sets for the sensors for both train car data and semi-truck data can be found in the Training Sets subsection. Information on embedded platforms used as edge devices is included in the Embedded Platforms subsection. A brief description of several metrics of interest is provided in the final subsection within Materials and Methods. The performance of the networks executed on both training PCs and embedded platforms is presented with various metrics in the Training Results and Embedded Platform Results subsections, respectively, within the Results section. The Discussion section covers general outcomes of the research, as well as future works.

## 2. Related Works

Neural networks are commonly used for performing both object detection and semantic segmentation [17,18,19,20]. Object detection is the process of identifying objects within an image, often times placing axis-aligned bounding boxes around the objects [21,22,23,24]. Semantic segmentation more specifically categorizes each pixel of an image as belonging to an object of interest or not [25,26,27]. To perform semantic segmentation, several types of deep neural network models can be utilized [28,29,30,31,32]. These include CNNs [33,34], Graph Neural Networks (GNNs) [35], and transformers [36,37], among others. However, CNNs are among the most commonly used methodologies due to the effectiveness of the convolution layer at extracting image features [33]. As such, there are many publicly available tools that have been developed in order to train a CNN for semantic segmentation [38].

Among these tool sets are a diverse range of network architectures available for utilization, including Multi-Receptive Field Module (MRFM) [39], Pyramid Scene Parsing Network (PSPNet) [40], Fully Convolutional Networks (FCN) [41], and DeepLabV3+ [42], among many others. With the wide range of networks available, it can be difficult to determine which to choose for a certain application. A common selection process is to evaluate each network’s performance on common, publicly available datasets, such as the PASCAL Visual Object Classes (PASCAL-VOC) dataset [43]. DeepLabV3+ was the best performing network at the time of network selection [44]. DeepLabV3+ is an extension to the original DeepLabV3 network architecture, and utilizes Atrous Spatial Pyramid Pooling (ASPP) in order to perform convolution. However, DeepLabV3+ also incorporates the common Encoder–Decoder methodology in order to improve segmentation speed and performance. DeepLabV3+ also allows for the utilization of multiple different backbones to make up the network architecture, including XCeption [45], RESNet [46], and MobileNetV2 [47]. These various backbones impact how many parameters the network needs to train, impacting both accuracy and speed.

There have been many analyses of LiDAR and camera sensor fusion approaches for a semantic segmentation application [8,48,49,50,51,52]. However, it is much harder to find a strict comparison of camera vs. LiDAR performance on semantic segmentation of the same dataset. This is, in part, due to the fact that cameras and LiDAR are inherently different sensor modalities with different resulting data formats; a 3D point cloud scan cannot be directly compared against a 2D image. For both fusion techniques and comparison studies, LiDAR data are usually converted to a range image, and this results in some amount of data loss. The semantic segmentation performance results between different modalities is useful for when only one type of sensor can be used. Inherently, LiDARs and their associated point clouds offer several advantages over traditional camera-based systems [48,53]. LiDAR is not dependent upon ambient lighting conditions, and it can be very robust when compared specifically to cameras in low-light conditions [54,55,56,57,58]. LiDAR also provides distance data directly as an output from the sensor, while camera-based systems typically require significant processing to produce distance data for each pixel [59,60].

Several methods exist to process point clouds using CNN models. First, the point cloud can be voxelized and a 3D CNN used to process the input [61]. This method is extremely computationally intensive and the majority of the compute time is wasted due to the sparsity of the point cloud. The Bird’s-Eye-View method is less intensive, but it is inefficient on embedded platforms [62]. A spherical projection is less computationally expensive and more suitable for converting point clouds to an image representation on the edge in real-time [63]. This is a critical step that is required to be able to compare image and LiDAR segmentation results against one another.

## 3. Materials and Methods

### 3.1. Dataset

The utilization of the UA_L-DoTT dataset was crucial in comparing the LiDAR vs. camera performance for semantic segmentation in varying locations under different weather conditions [16]. This multimodal dataset consists of large amounts of annotated data from five unique LiDAR—Velodyne Puck (VLP-16), Velodyne Puck Hi-Res (VLP-16 Hi-Res), Velodyne 32MR, Ouster OS-1, and Ouster OS-2—as well as a 2D RGB monocular camera—FLIR Blackfly S GigE BFS-PGE-31S4C-C. The dataset contains approximately 93,000 raw images and 350,000 raw LiDAR scans, collected across four separate locations with varying environmental conditions. This further allowed evaluation of the presented system to generalize to a range of environments. The accompanying dataset includes all of the raw images and point clouds collected, as well as 9000 labeled text files that correspond to the images, 77,000 labeled point clouds, and 33 timestamp files. The timestamps correlate images to point clouds via POSIX time. The dataset is publicly available at UA_L-DoTT (https://plus.figshare.com/articles/dataset/UA_L-DoTT_University_of_Alabama_s_Large_Dataset_of_Trains_and_Trucks_-_Dataset_Repository/19311938/1, accessed on 15 March 2022) [64].

Data collection was performed during five separate instances, four of which targeted train tracks (with one repeat location) and one that targeted a four-lane highway (for semi-truck targets). The distance to the targets of interest ranged from 42 to 240 m, depending on the site. The base data collection frequency for the VLP-16, VLP-16 Hi-Res, and 32MR was 8 Hz, and the baseline for the OS-1, OS-2, and Blackfly was 10 Hz. Only every 3–7 images were labeled, or roughly data at 1–3 Hz, to increase the diversity of data. LiDAR downsampling also occurred at several of the sites, resulting in labeling every 3–5 scans, or a labeling frequency of 1–3 Hz, to ensure adequate change in scenery across consecutive scans. Lighting conditions also ranged from bright sun to overcast to darkness. Data were collected statically at the first four sites. Here, the stationary sensors were panned back and forth occasionally to vary the viewpoint while maintaining a set distance between the sensors and targets of interest. Readers interested in additional detailed information regarding the creation and characteristics of the dataset are directed to [16]. During the final data collection, the sensors were mounted to a dynamic vehicle to provide a constantly changing viewpoint while maintaining a similar distance between the sensor setup and the targets of interest. Although it is expected that real-world sensor deployments will be roadside and stationary, collecting data from a mobile platform helped create a more robust training set, requiring the networks to identify the targets regardless of their positions within the sensor’s FoV. A specific site location will be known before deployment but, critically, the exact sensor location may or may not be known. The distance from the targets of interest is an important parameter that must be defined when deploying the roadside sensor suite to ensure adequate coverage. However, the sensors may be deployed anywhere along a roadside at this set distance to avoid occlusions or obstacles.

### 3.2. Sensor Characteristics

RGB cameras and LiDARs represent data in fundamentally different formats. LiDAR is a time-of-flight technology that emits a laser pulse and measures the time it takes to receive the echo after the laser pulse reflects off various objects in the field-of-view (FoV). LiDARs are available in “spinning” models and solid-state models. Spinning models use an emitter and a detector (or a set of them) that spin completely around the vertical axis, providing a $360^{\circ }$ horizontal FoV. There are several characteristics of a spinning LiDARs that directly impact the point clouds produced. The horizontal FoV is typically $360^{\circ }$, but the vertical FoV varies depending on the model. Specifically, the number of scanlines and the vertical angular resolution define the vertical FoV and the vertical point density.

Images collected from an RGB camera, as well as point clouds from multiple spinning LiDARs with varying operational characteristics, are utilized for identifying vehicles of interest in this work. Inherently, LiDARs and their associated point clouds offer several advantages over traditional camera-based systems. For example, LiDARs are not dependent upon ambient lighting conditions. Secondly, spinning LiDARs have a $360^{\circ }$ horizontal FoV, significantly larger than a standard 2D camera. This can reduce the number of deployment units necessary to cover the region of interest. LiDARs provide distance data directly as an output from the sensor while cameras typically require significant processing to produce distance data for each pixel. This additional data allows users to take advantage of the spatial relationships among objects in the FoV, minimizing the complexity required to count target instances. Table 1 shows the sensor specifications for the five LiDAR sensors used in this project. Synced data were collected from these sensors at each of the five data collection sites. This provided slightly different-looking data all targeting the same scene, allowing a comparison of the data generated from each of the LiDAR. For example, some LiDARs were significantly cheaper than others, but had a shorter range. This meant that not all targets of interest were recognized by all LiDARs if they were past a certain distance. Similarly, a LiDAR with a larger vertical or horizontal resolution might miss smaller targets far away. The LiDARs were chosen to provide variety in number of scanlines (S.Lines), vertical FoV (V. FoV), vertical resolution (ω), horizontal resolution (α), and range. We draw the reader’s attention to the range metric. LiDAR range is a function of the intensity and size of the target and, in most cases, the provided range metric is measured using targets of varying color, size, and reflectivity. Therefore, the stated ranges are often not consistent in real-world operations.

### 3.3. Camera Architecture

Segmentation of the 3D point cloud data and 2D image data are treated as two independent tasks. Many off-the-shelf models exist to perform image segmentation. We chose the DeepLabV3+ network architecture for the reasons discussed in Section 2. The MobileNetV2 backbone is optimized to run on embedded devices, as would be needed in edge deployments, so it was integrated into the network [47]. Transfer learning with the PASCAL-VOC dataset was utilized to accelerate the training process [44].

The model was trained using the Adam optimizer with the Categorical Cross-Entropy loss function [65,66]. This enabled high quality results with minimal hyperparameter optimization. Evaluation was performed using the mIOU and standard deviation metrics. Standard augmentations including Gaussian noise, random flipping, and random crops were applied to reduce overfitting.

### 3.4. LiDAR Architecture

A spherical projection was chosen to convert the point clouds to an image representation due to its efficiency on embedded platforms. This system converts the Euclidean representation of point clouds into spherical coordinates which are then projected onto an image plane. The resulting resolution is the number of scanlines times the number of horizontal points per scanline. Each pixel in the image includes the point coordinates, (XYZ), and the return intensity, I. The input to the network is therefore an image with four input channels (XYZI) instead of three colors (RGB). The model then outputs (N, H, W, C) tensors, where N is the point cloud index, H and W are the pixel coordinates, and C is the confidence associated with the particular class, vehicle or not a vehicle, normalized with softmax.

This projection produces varying image dimensions dependent upon the number of scanlines per LiDAR. Thus, LiDARs with different numbers of scanlines require separate deep learning models. It is advantageous to have one, unified model capable of accepting point clouds from all the LiDAR scanners. For this reason, a nearest-neighbor up-sampling methodology to increase the scanlines of the lower resolution LiDARs was developed. Each scanline of the lower resolution LiDAR is duplicated such that the height of the final input image remains equal to the maximum number of scanlines that are supported.

DeepLabV3+ utilizes symmetric down-sampling of the input data, which works well for relatively square input data, such as camera imagery. However, the vastly asymmetric point cloud resolution (16 × 2048) would result in poor segmentation performance of the network. When the input data only has 16 rows, downsampling quickly decimates the resolution of the data. This causes the model to lose critical spatial information used to produce the semantic segmentation. To address this, an architecture inspired by DeepLabV3+ was created to downsample exclusively across the width of the input data. Additionally, the dilations in the atrous kernels are applied only along the width. This enables the use of an architecture very similar to DeepLabV3+ without the internal decimation of the point cloud resolution. The point cloud model follows the same training process as the camera model. Again, categorical cross-entropy loss, the Adam optimizer, and the mIOU/standard deviation evaluation metrics are used. Random flipping, Gaussian noise to the range of the return, and Gaussian intensity noise augmentations are applied during training.

### 3.5. Training Sets

Numerous benchmarks of the presented systems were tested and compared between sensors. Many of these benchmarks required a different training set for accurate evaluation. An initial split of all data were dictated by the target of interest: train car, discussed in the sections below, or semi-truck, discussed in Appendix A. A summary of the various testing sets is outlined in Table 2 for the train car dataset below and Table A1 for the truck dataset in the Appendix. In each table, “Pos. Scans” refers to any scan in the dataset that included a target of interest, and “Neg. Scans” refers to any scans that did not. The datasets are split into training, “Train”, validation, “Val.”, and test, “Test”, sets. These sets were randomly generated, and consisted of 80%, 10%, and 10% of the total size of the overall data, respectively. Due to randomization during the generation of training, validation, and test set splitting, similar scans may appear across sets. This results in the models intentionally overfitting to data at these specific locations. Commonly, overfitting is viewed as a negative byproduct of not following proper training procedures. However, we show an alternative, beneficial use case for overfitting when inference is always performed on very similar data in the same “scene”. In a real-world deployment, sensor(s) would be placed in a stationary position targeting a railway or road of interest. Here, the viewpoints of the targets would not change, opposite to a constantly changing viewpoint used in autonomous vehicles, and this static relationship can be exploited to increase detection accuracy at these specific deployment locations with a priori knowledge of FoV and target perspective.

The Puck, Hi-Res, 32MR, OS-1, and OS-2 sets were utilized to test how the network performed when trained and tested on data from a single sensor. This served as a baseline in the nearest-neighbor up-sampling methodology previously discussed. The Camera set was produced to evaluate the overall performance compared to the LiDAR sets. The All-LiDAR set was generated to see how well the presented system generalized to any network with all LiDAR sensors. This allows testing of a single, unified model independent of sensor parameters.

The “Light (LiDAR)” and “Light (Camera)” sets were produced to compare the low-light performance of the proposed LiDAR system to that of a camera-based system. Only data from the 32MR was utilized in this LiDAR set as a mid-range sensor. Utilization of a single sensor ensures any issues from the up-sampling methodology does not negatively impact this benchmark. Both the low-light LiDAR and camera sets were generated utilizing data collected at site 4. Finally, “Gen. (LiDAR)” and “Gen. (Camera)” are generalization sets utilized to evaluate the capability of the proposed system to perform in environments not seen during the training process. Here, the up-sampling methodology was utilized with all LiDAR. If the unified model performs comparably to the individual models, then its generalization performance can reasonably be extrapolated to the individual models. Generalization sets were generated using sites 2, 4, and 5, and then inferred and evaluated on site 1. This allows the only variable of change to be the environmental characteristics of the location.

### 3.6. Embedded Platforms

The models were normally trained on lab PCs containing high-end hardware including NVIDIA Quadro RTX8000 or Titan RTX GPUs. However, embedded platforms such as the NVIDIA Jetson family have special hardware intended for running deep learning models in real-time. The NVIDIA Jetson AGX Xavier, Nano, and NX as well as a Raspberry Pi 4 outfitted with a Google Coral Tensor Processing Unit (TPU) were evaluated for computing on the edge.

### 3.7. Metrics of Interest

There are a multitude of metrics used to measure performance in the world of segmentation prediction. A brief description of common metrics and justification for using each is provided below. Most of the metrics are derived from four variables:

1. True Positive (TP)—the ground truth “positive” label for a given class;
2. True Negative (TN)—all other parts of the data that are not the class label;
3. False Positive (FP)—the predicted portion of an object that is not labeled as that class in the ground truth data;
4. False Negative (FN)—the ground truth object that is not classified in the prediction.

Using these four variables, several different performance metrics can be defined for evaluating inference results against the ground truth data. The first is recall which looks at the correctly labeled class TP versus the remainder of the ground truth class FN,

$$Recall=\frac{TP}{TP+FN}$$

**Figure 2:**
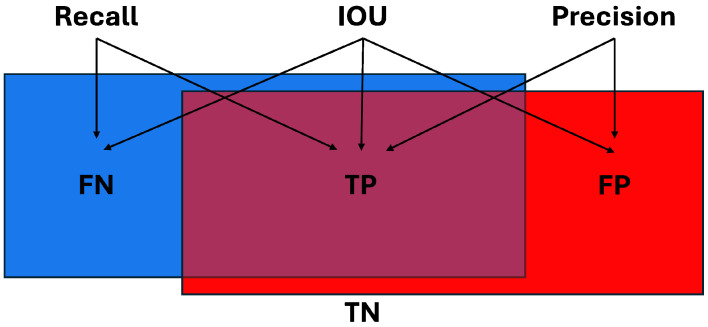
Example of ground truth train car represented in blue, predicted train car highlighted in red, and overlap between the two shown in purple. Different regions of the prediction are labeled with TP, TN, FP, and FN. Note that IOU is dependent on TP, FN, and FP, whereas recall is only dependent on TP and FN, and precision is only dependent on TP and FP.

This is useful when determining what portion of the entire ground truth class was labeled correctly, but does not provide any information on how much data were incorrectly labeled as a class, highlighted in Figure 2. Precision, also shown in Figure 2, evaluates what percentage of the estimated output was correct by looking at TP and FP,

$$Precision=\frac{TP}{TP+FP}$$

Precision identifies what portion of the estimated output was correctly labeled, but disregards any ground truth label that was missed. Accuracy considers all four variables to attempt to obtain the full picture of the predicted result,

$$Accuracy=\frac{TP+TN}{TP+TN+FP+FN}$$

However, TN can throw off results if its quantity is high, as this artificially inflates the accuracy. By removing TN from the equation, IOU can provide a more realistic accuracy metric that evaluates all parts of the data,

$$IOU=\frac{Intersection}{Union}=\frac{TP}{TP+FP+FN}$$

Notice in Figure 2 that IOU evaluates all parts of the data that are labeled as a specified class in both the ground truth and prediction. Another metric that evaluates TP, FP, and FN variables is F1. It combines precision and recall together resulting in a very similar equation to IOU,

$$F1=\frac{2\times Precision\times Recall}{Precision+Recall}=\frac{2\times \frac{TP}{TP+FP}\times \frac{TP}{TP+FN}}{\frac{TP}{TP+FP}+\frac{TP}{TP+FN}}=\frac{TP}{TP+0.5(FP+FN)}$$

The primary difference between the two most recent metrics mentioned is that F1 places half as much of an emphasis on FP and FN as IOU does. This results in a metric that rewards TP more and punishes FP and FN less. We believe that IOU better captures the accuracy of the system, and thus use mIOU to measure accuracy across all pixels/points. Another metric utilized is the standard deviation, σ, of mIOU,

$$\sigma =\sqrt{\frac{\sum_{i=1}^{n}{\left(IOU\left(i\right)-\bar{IOU}\right)}^{2}}{n-1}}$$

where *n* is the number of images/point clouds, IOU(i) is the IOU score for a given image/point cloud, and $\bar{IOU}$ is the average mIOU score across all images/point clouds. The standard deviation helps account for uncertainty and variability in the prediction performance to identify how reliable the network behaves. The final metric utilized is frames per second (FPS) to evaluate whether the deployed network classifies as a real-time system.

## 4. Results

The segmentation performance results are broken down into Training Results and Embedded Platform Results subsections. The Training Results are further split into Individual Sensors, Sensor Generalization, and Environmental Generalization. The Individual Sensors subsection covers the validation mIOU, testing mIOU, testing standard deviation, and frame rate metrics of the individual sensor datasets. The Sensor Generalization subsection covers the same metrics of the All-LiDAR datasets. The Environmental Generalization subsection covers the same metrics of the generalization and low-light datasets. The Embedded Platform Inference Results discusses results read from all embedded platforms. Each of the various subsections within Segmentation Performance Results presents the performance related to train car targets. The performance for truck targets is presented in the Supplementary Truck Results (Appendix A) using the same general format.

### 4.1. Training Results

The validation mIOU, testing mIOU, and standard deviation performance metrics for all train car based evaluation sets are outlined in Table 3. The validation mIOU was used in the training process to identify and save the weights of the best model. Testing mIOU is calculated on new data not seen during the training process, and is therefore more representative of how the network will perform in the field. As should be anticipated, the testing mIOU is slightly lower than the validation mIOU for all of the datasets presented. The testing standard deviation captures some of the edge cases within these inferences. It also gives some insight into how often FP or FN may be output by the network.

#### 4.1.1. Individual Sensors

The mIOU data for the individual LiDARs are better than those for the camera model. This is a general indication that LiDAR may be better suited for this task. The drop from validation mIOU to testing mIOU is also smaller for these individual LiDAR models. This indicates the LiDAR-based models are more capable of learning the features of an object of interest. A low standard deviation of the camera model indicates that the camera-based system results in few FP and FN when inferring on new data. The OS-1 standard deviation is lower than its counterparts; however, it is believed that this is a function of the training dataset composition and less likely an indication of better performance. The respective frame rates of the individual sensor models are around twice the collection frequency used by the LiDAR (8–10 Hz) and camera (10 Hz), indicating the system is able to perform real-time on high-end hardware.

**Figure 3:**
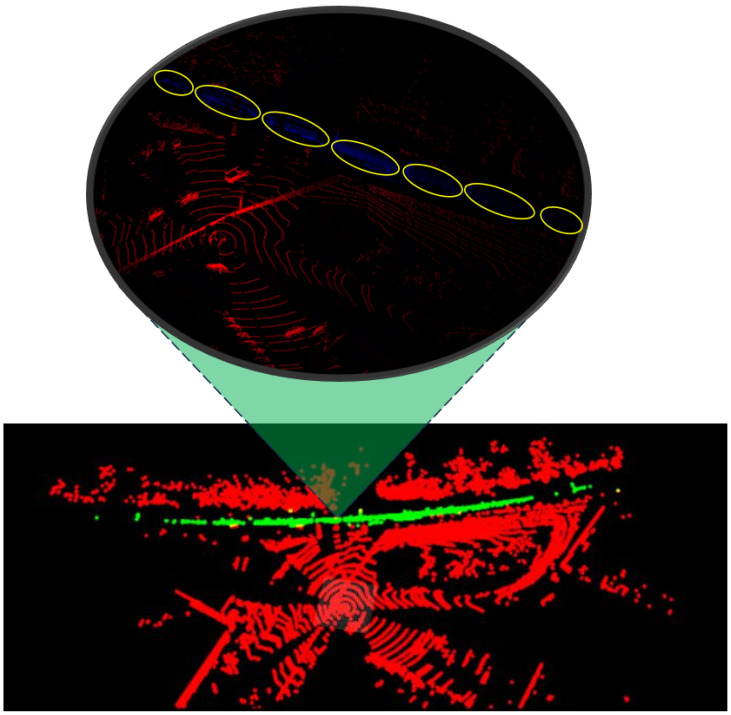
Train car 32MR model output.

Figure 3 shows the LiDAR segmentation model output for the 32MR train car data. The individual train cars are circled in yellow for ease of visualization. While the network reports a confidence score of the class for each individual point in the cloud, any point with a positive score is represented as green (object of interest), and negative scores as red (background).

**Figure 4:**
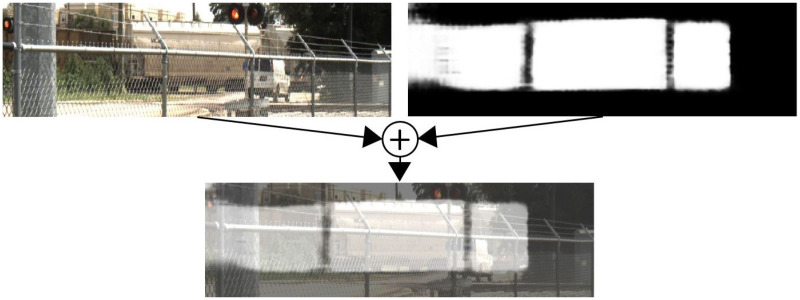
Overlay of raw camera image and network mask.

Figure 4 shows a raw camera image in the top left corner and the associated network output mask from the testing set in the top right corner. The mask represents the target identification performed by the camera model. White pixels indicate points the model identified as part of a train car, and black pixels are considered not part of the train car. The output mask overlaid with the raw image is shown in the bottom of the figure for ease of visualization.

#### 4.1.2. Sensor Generalization

**Figure 5:**
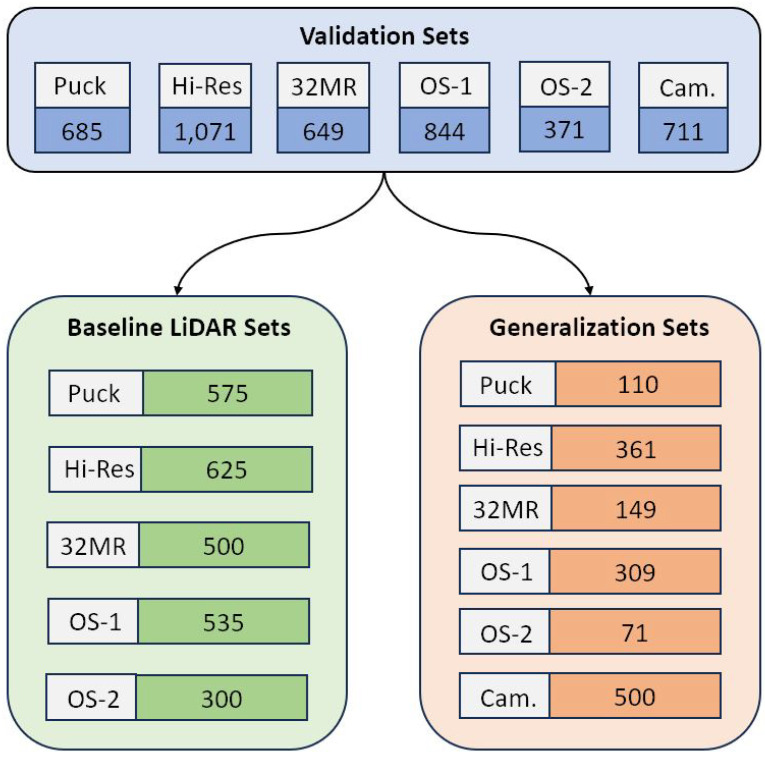
Baseline and generalization set split.

All LiDAR data were merged into a single dataset to create a generic all-LiDAR model. To fully validate the all-LiDAR methodology, this generic model was inferred using validation sets generated prior to any dataset creation. This ensured that no individual sensor was trained on data it was tested on. The validation sets were split into separate baseline LiDAR and generalization sets, visualized in Figure 5. First, the individual sensor models were used to infer upon the corresponding sensor-specific baseline validation set, and the testing mIOU and standard deviation were found. This serves as a baseline for the individual sensors shown in Table 4. Then, the generic All-LiDAR model was used to infer upon the same sensor-specific baseline data.

Table 5 shows the results of the All-LiDAR model using the same test sets that the individual LiDAR baselines used in Table 4. This model ultimately utilized every labeled scan available in the UA_L-DoTT dataset. The mIOU and standard deviation values of the generic All-LiDAR model, shown in Table 5, are nearly identical to the performance of the individual LiDAR models. This indicates that merging these sensors together into a single, generic LiDAR model does not sacrifice performance.

**Figure 6:**
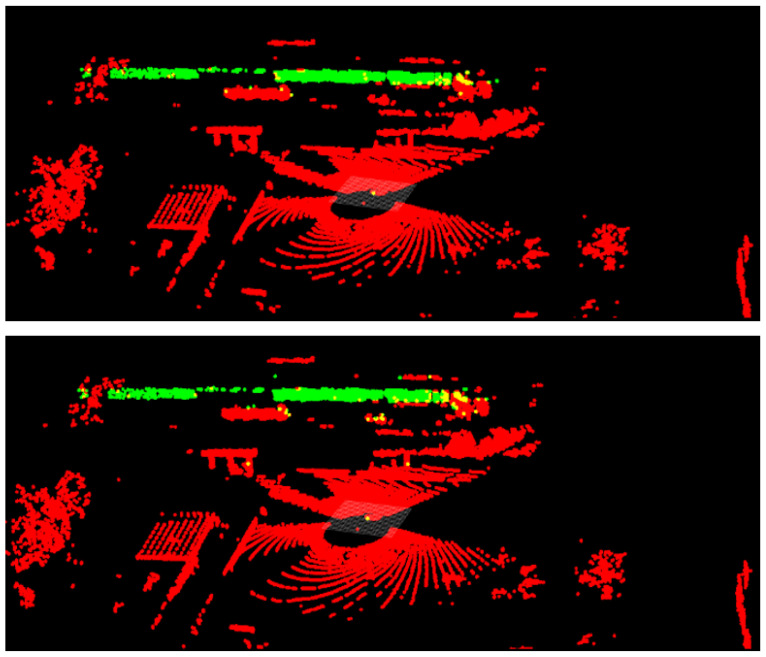
The OS2 baseline results are shown on the top. The All-LiDAR results on the same scan are shown on the bottom with very similar performance.

A visual representation of the similarity of the performance between the baseline LiDAR sets and the All-LiDAR set is shown in Figure 6. The top of the figure is the 32MR baseline result vs. the bottom of the figure which is the All-LiDAR baseline result. The differences between the two are almost indistinguishable.

#### 4.1.3. Environmental Generalization

An additional benefit that LiDAR have over camera-based systems is their indifference to ambient lighting conditions. The low-light performance during training and testing is shown in Table 3. These models were then executed on validation datasets composed of low-light camera images and LiDAR scans. This shows how these models generalize to low-light conditions and compares their performance. Table 6 shows the results of this generalization to low-light conditions. As expected, the LiDAR model significantly outperforms the camera system in this low-light environment.

The performance of the models on the generalized training datasets is shown in Table 3. The generalization models were trained on train car data from sites 2, 4, and 5. Prior to any dataset generation, 500 images and 1000 point clouds from site 1 were set aside. Then, the LiDAR and camera models ran inference on these data from a separate scene, site 1, not used during the training process. All other training datasets were designed to increase accuracy at one specific site, e.g., intentional overfitting, but the trade-off is this reduces the generalization capacity of the models. This is shown with the overall reduced performance in Table 7. Although the other models performed exceptionally well, when tasked with generalization to a previously unseen scene, the generalization models take a significant dip in performance. The LiDAR model’s decreased performance over the camera model is likely a function of the limited number of data points in each scan that hit the target(s) of interest compared to the number of pixels in a camera image that “see” the target(s). Even with only half of the data used for the generalized LiDAR set, the generalized camera performance is 11.48% more accurate with a 15.12% lower standard deviation. This higher accuracy and significantly lower rate of FP indicates that the camera model may be initially better suited to deployments in completely new environments when data is limited.

**Figure 7:**
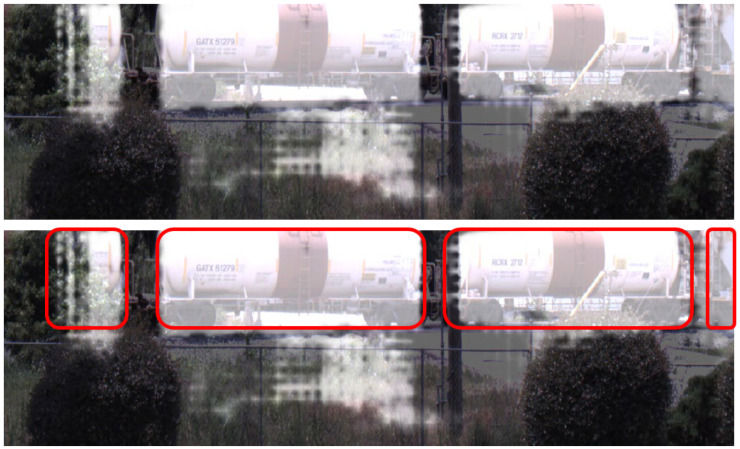
Overlay of raw camera image and network mask during environmental generalization on site 1 on top. The bottom half is the same image with the train cars hand labeled for ease of visualization.

**Figure 8:**
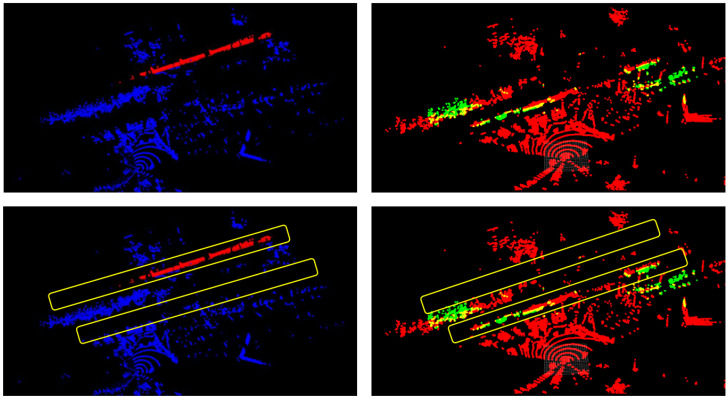
All-LiDAR performance during environmental generalization on site 1. The top left corner is the hand labeled scan with the background represented with blue points and the train cars represented with red points. The top right corner shows the inference results with background represented with red points and the train cars represented with green points. The two bottom corners are the top images with hand labeled areas of the two tracks in yellow for ease of visualization.

An example of the inference output of the camera model run on the generalized site 1 data are shown in Figure 7. All train cars, circled in red for visualization, are correctly segmented, but extra pixels beneath the train cars are incorrectly labeled as the vehicle class. The LiDAR model has a poorer performance. An example is displayed in Figure 8, where the top left figure is the hand labeled scan where the blue points represent the background, and the red points indicate the train car. An inference result is shown in the top right figure, where red points represent the background and green are the train car. Site 1 contained two tracks, represented by the two yellow rectangles in the two bottom figures. In the hand-labeled scan, the train car points run directly along the track. However, the inference results only contain some points within the track, mistaking many of the brush and tree points in the background to be a train car. The quantitative and qualitative results both point towards the current LiDAR model requiring additional training to become more robust and site agnostic. This additional, wider range of training data would likely increase performance across locations.

### 4.2. Embedded Platform Inference Results

In order to measure the ability of the model to run in real-time on the edge, we selected inference time as our primary metric. In cases where the mIOU score on the embedded device was the same as the lab computer, it has been omitted from this section. The NVIDIA Xavier and NX platforms run the exact same models executed on the training PCs, resulting in equivalent performance. The mIOU scores for the TPU models are reported; however, as they are substantially different than lab PC results. Table 8 shows the performance of the train car datasets across the embedded platforms. “CNR” means “Cannot Run” and indicates a model was unable to execute on an embedded platform. The LiDAR-based model was unable to execute on the Jetson Nano due to an inadequate amount of RAM. The camera model was unable to execute on the TPU due to unsupported operations such as the softmax function.

To run inference on the TPU, the models were converted to a fully quantized int8-based version. This was accomplished with post-training quantization so the results from training could be utilized on any embedded platform. These changes resulted in considerable reductions in performance, with the mIOU ranging from 51.93% to 67.58%, compared to a range of between 90.89% and 99.86% on the lab PCs and other edge devices. The standard deviation is also marginally higher for each LiDAR except for the OS-2. While the camera-based model has a faster frame rate than the LiDAR models on the training PCs, it is slower than the LiDAR models on the other embedded platforms. Each LiDAR model operates at approximately 7.4 FPS on the Xavier, and 9 FPS on the NX. As such, each LiDAR tested operated in real-time. These models include no platform-specific optimizations, and could be improved. The performance represents worst-case and improvement is possible using various TensorRT optimizations.

## 5. Discussion

The proposed roadside detection system has the potential to be a powerful tool in tracking vehicles of interest along any road. Targets of train cars and semi-trucks were successfully classified in multiple environments with changing distances from the sensors and traveling at varying speeds. Both LiDAR and camera data were successful at segmenting the targets from other vehicles traveling along the same roads with different advantages. In general, the LiDAR models had comparable validation and testing mIOU results to the camera models. They also displayed a slightly higher frame rate on the embedded platforms. However, the results indicate that the LiDAR models do not generalize as well as the camera models, and thus could require more training to obtain comparable performance when deployed to new sites. Generic LiDAR models such as the All-LiDAR set can be developed to perform well across different LiDAR sensors. This could lead to sensor agnostic deployments that have many benefits.

The strong performance of LiDAR for this application points toward it being a viable and powerful alternative to cameras for semantic segmentation and object classification on the edge. Excellent vehicle semantic segmentation results with data from various vision sensors can be achieved with the proposed roadside/trackside perspective in real-time. The preliminary results indicate this method as being a useful tool to detecting vehicles such as train cars or semi-trucks along any roadside in a non-intrusive manner. This is especially useful in rural areas and back roads where there may be no other intelligent transportation systems present. One specific commercial application is for the detection of semi-trucks on small roadways attempting to bypass required weigh stations which are typically located on primary or major traffic routes. The transportability of this system lends itself to deployment and redeployment for this application and can offer states a solution to this growing problem [67,68,69]. Similarly, this system is capable of remote deployment trackside to monitor railroad traffic for verification of payloads. In urban areas, several upgrades could be integrated into the system. Multiple roadside sensor deployments could remotely connect to one another for identifying heavy flow paths of semi-trucks. Additionally, pre-existing intelligent transportation systems could utilize these data for: (1) counting the instances of vehicles of interest for traffic management; (2) estimating the flow of payloads or personnel of interest along a certain route; (3) rerouting traffic to avoid congestion.

Future works include increasing the robustness of the roadside detection system. The LiDAR models perform well in a low-light environment, but more challenging, real-world conditions such as heavy rain or snow could be difficult for LiDAR to take accurate readings due to unwanted reflections. Performing sensor fusion between the camera and LiDAR could increase the resilience of the system in these extreme weather situations. This will require restructuring the CNN architecture to accept and correlate multimodal raw data that will be fused together through a multi-channel input. More data with a wider range of weather conditions and locations must be collected and trained on to further strengthen the models. Effort will be made to collect smaller amounts of data at a larger number of data collection sites to enhance the diversity of the system and increase the immediate usability. Finally, the models must be tested with other publicly available datasets such as Cityscapes [70] to perform a comparison study. Similarly, state-of-the-art models will be tested with the custom dataset in future studies to further evaluate the effectiveness on other models.

## Figures and Tables

**Table 1:** LiDAR models and Characteristics: number of scanlines (S.Lines), range, accuracy (Acc.), horizontal FoV (H. FoV), horizontal angular resolution (α), vertical FoV (V. FoV), vertical angular resolution (ω), and power consumption for the Velodyne VLP-16, Velodyne VLP-16 Hi-Res, Velodyne 32MR, Ouster OS-1, and Ouster OS-2 spinning LiDARs are shown.

	VLP-16	VLP Hi-Res	32MR	OS-1	OS-2
S.Lines	16	16	32	16	64
Range	100 m	100 m	120 m	120 m	240 m
Acc.	±3 cm	±3 cm	±3 cm	±1.5–10 cm	±1.5–5 cm
H. FoV	$360^{\circ }$	$360^{\circ }$	$360^{\circ }$	$360^{\circ }$	$360^{\circ }$
α	$0.1^{\circ }$–$0.4^{\circ }$	$0.1^{\circ }$–$0.4^{\circ }$	$0.1^{\circ }$–$0.4^{\circ }$	$0.18^{\circ }$–$0.7^{\circ }$	$0.18^{\circ }$–$0.7^{\circ }$
V. FoV	$-15^{\circ }$–$15^{\circ }$	$-10^{\circ }$–$10^{\circ }$	$-25^{\circ }$–$15^{\circ }$	$-22.5^{\circ }$–$22.5^{\circ }$	$-11.25^{\circ }$–$11.25^{\circ }$
ω	$2^{\circ }$	$1.33^{\circ }$	$0.33^{\circ }$ min.	$3^{\circ }$	$0.36^{\circ }$
Power	8 W	8 W	10 W	14–20 W	14–20 W

**Table 2:** LiDAR and camera training sets for train car evaluation.

Training Set	Pos. Scans	Neg. Scans	Train Size	Val. Size	Test Size
Puck	6229	623	5481	685	686
Hi-Res	10,037	672	8567	1071	1071
32MR	5899	590	5191	649	649
OS-1	7673	767	6752	844	844
OS-2	3377	338	2972	371	372
Camera	6466	647	5690	711	712
All-LiDAR	33,215	3322	29,229	3654	3654
Light (LiDAR)	4468	0	3574	447	447
Light (Camera)	5830	529	5087	636	636
Gen. (LiDAR)	28,646	2865	25,208	3151	3152
Gen. (Camera)	3333	333	2932	367	367

**Table 3:** Train car mIOU results.

Dataset	Validation MIOU	Testing MIOU	Testing StdDev	FPS
Puck	99.78%	99.14%	4.96%	18.476
OS-1	99.94%	99.86%	0.90%	18.696
Hi-Res	99.92%	99.30%	4.49%	18.636
32MR	99.85%	99.41%	3.34%	18.243
OS-2	99.36%	98.27%	6.19%	17.084
Camera	93.68%	90.89%	0.34%	21.716
All-LiDAR	99.79%	99.15%	5.07%	17.015
Low Light 32MR	99.88%	98.80%	6.79%	N/A
Low Light Camera	93.66%	92.01%	0.36%	N/A
General. LiDAR	99.80%	99.20%	4.85%	N/A
General. Camera	92.43%	90.66%	0.56%	N/A

**Table 4:** LiDAR baselines.

Set	Set Size	Testing MIOU	Testing StdDev
Puck Baseline	575	99.28%	3.37%
Hi-Res Baseline	625	99.35%	4.07%
OS-1 Baseline	535	99.59%	3.17%
32MR Baseline	500	99.21%	3.56%
OS-2 Baseline	300	98.79%	3.29%

**Table 5:** LiDAR generalization performance.

Set	Set Size	Testing MIOU	Testing StdDev
All-LiDAR on Puck	575	99.84%	0.84%
All-LiDAR on Hi-Res	625	99.52%	3.09%
All-LiDAR on OS-1	535	99.34%	5.12%
All-LiDAR on 32MR	500	99.10%	4.56%
All-LiDAR on OS-2	300	98.34%	3.01%

**Table 6:** Train car low-light generalization performance.

Set	Set Size	MIOU	StdDev
Low Light 32MR	447	98.31%	1.55%
Low Light Camera	636	34.23%	1.04%

**Table 7:** Train car generalization performance.

Set	Set Size	MIOU	StdDev
General. LiDAR	1000	61.93%	16.33%
General. Camera	500	73.41%	1.21%

**Table 8:** Train car inference embedded platform results.

Dataset	Xav. FPS	Nano FPS	NX FPS	TPU MIOU	TPU S.D.	TPU FPS
Puck	7.090	CNR	8.830	67.58%	10.98%	2.12
OS-1	7.311	CNR	9.266	58.01%	5.80%	2.12
Hi-Res	7.567	CNR	9.590	64.07%	9.42%	2.08
32MR	7.516	CNR	8.897	52.29%	5.43%	1.04
OS-2	7.532	CNR	8.586	51.93%	3.06%	0.49
Camera	6.235	5.257	7.741	CNR	CNR	CNR
All-LiDAR	7.641	CNR	9.591	55.36%	5.90%	0.49

**Figure A1:**
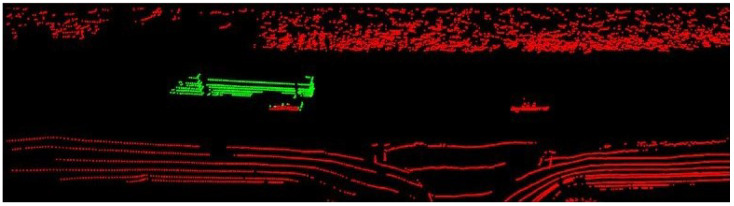
32MR truck inference. There are two non-target pick-up trucks and one target semi-truck. The entirety of the semi-truck is correctly labeled, and the pick-up truck in front is partially mislabeled as part of the semi-truck.

**Figure A2:**
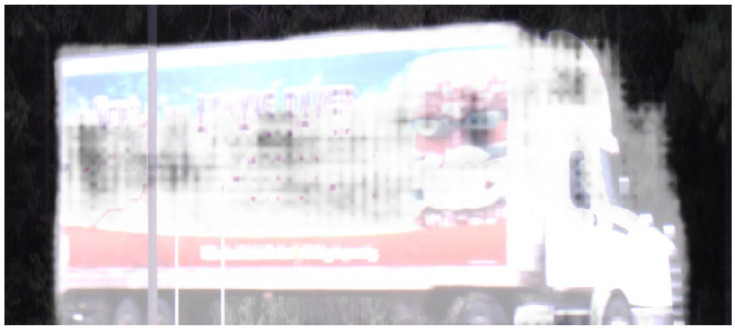
Camera truck inference. The entirety of the semi-truck is correctly labeled, but the area directly in front is mislabeled as a false positive.

## Data Availability

A publicly available dataset was analyzed in this study. These data can be found here: UA_L-DoTT (https://plus.figshare.com/articles/dataset/UA_L-DoTT_University_of_Alabama_s_Large_Dataset_of_Trains_and_Trucks_-_Dataset_Repository/19311938/1, accessed on 15 March 2022) [64].

## Appendix A. Supplementary Truck Results

A summary of the various testing sets is outlined in Table A1 for the truck dataset. The training and testing results for the truck models of each sensor are then shown in Table A2. The data collected by the OS-1 at the truck site was at the limit of its range and resulted in poor scan quality, so it was omitted. The results of the remaining LiDAR are generally poorer than for the train car model. The large drop in mIOU from the validation to the testing set indicates that the camera-based model may be suffering from lack of diverse training data. This is a function of collecting truck data from only one field site, and it is expected that additional training data would lessen this problem.

**Table A1:** LiDAR and camera training sets for truck evaluation.

Training Set	Pos. Scans	Neg. Scans	Train. Size	Val. Size	Test. Size
Puck	1604	160	1411	176	177
Hi-Res	2015	202	1773	222	222
32MR	2268	227	1996	249	250
OS-2	1528	153	1344	168	169
Camera	2804	280	2467	308	309
All-LiDAR	7415	548	6370	796	797

**Table A2:** Truck mIOU Results.

Dataset	Validation MIOU	Testing MIOU	Testing StdDev	FPS
Puck	93.17%	86.94%	14.19%	18.441
Hi-Res	98.52%	92.56%	15.52%	18.413
32MR	98.15%	96.02%	9.76%	18.285
OS-2	89.12%	86.89%	9.09%	17.146
Camera	97.38%	68.85%	1.55%	21.542
All-LiDAR	97.32%	95.33%	10.38%	17.431

Figure A1 shows two pick-up trucks in the same scan with a target semi-truck. The pick-up far to the right of the target is easily ignored by the model, but the pick-up next to the target is partially labeled as part of the target. This could be a function of the pick-up occluding parts of the semi-truck, or simply their close proximity. It is believed that this can be trained out of the model with additional instances of these types of scenarios.

An example of the camera performance is shown in Figure A2. Here, the entirety of the semi-truck is labeled correctly. However, there is a small grouping of pixels in front of the cabin that are incorrectly labeled as semi-truck. The training and testing results for the generic all-LiDAR truck model are also shown in Table A2. The overall performance reflects the limited training set. However, the performance of the generic all-LiDAR model is comparable to all the individual LiDAR truck models.

Each individual sensor model was ported to the various embedded platforms to execute inference. The same methodology used for the train car models was implemented. Table A3 shows the resulting performance. The mIOU and standard deviation for the NVIDIA platforms were the same as that for the training PCs. The performance of the individual sensor truck models on the embedded platforms is mostly consistent with that presented for the LiDAR train car models. The generic all-LiDAR model achieved a comparable frame rate on the NVIDIA platforms.

**Table A3:** Truck inference embedded platform results.

Dataset	Xav. FPS	Nano FPS	NX FPS	TPU MIOU	TPU S.D.	TPU FPS
Puck	6.947	CNR	9.719	67.42%	14.63%	2.10
Hi-Res	7.484	CNR	9.812	60.87%	8.80%	2.09
32MR	6.768	CNR	8.294	61.03%	11.38%	1.04
OS-2	7.440	CNR	9.272	59.28%	7.13%	0.50
Camera	6.574	4.812	7.298	CNR	CNR	CNR
All-LiDAR	7.302	CNR	8.614	54.21%	10.58%	0.50
